# Effects of Floral Scent, Color and Pollen on Foraging Decisions and Oocyte Development of Common Green Bottle Flies

**DOI:** 10.1371/journal.pone.0145055

**Published:** 2015-12-30

**Authors:** Bekka S. Brodie, Maia A. Smith, Jason Lawrence, Gerhard Gries

**Affiliations:** Simon Fraser University, Department of Biological Sciences, Burnaby, British Columbia, Canada; North Carolina State University, UNITED STATES

## Abstract

The common green bottle fly *Lucilia sericata* (Meigen) and other filth flies frequently visit pollen-rich composite flowers such as the Oxeye daisy, *Leucanthemum vulgare* Lam. In laboratory experiments with *L*. *sericata*, we investigated the effect of generic floral scent and color cues, and of Oxeye daisy-specific cues, on foraging decisions by recently eclosed flies. We also tested the effect of a floral pollen diet with 0–35% moisture content on the ability of females to mature their oocytes. Our data indicate that (1) young flies in the presence of generic floral scent respond more strongly to a uniformly yellow cue than to any other uniform color cue (green, white, black, blue, red) except for ultraviolet (UV); (2) the floral scent of Oxeye daisies enhances the attractiveness of a yellow cue; and (3) moisture-rich pollen provides nutrients that facilitate ovary maturation of flies. With evidence that *L*. *sericata* exploits floral cues during foraging, and that pollen can be an alternate protein source to animal feces and carrion, Pollen apparently plays a major role in the foraging ecology of *L*. *sericata* and possibly other filth flies. These flies, in turn, may play a significant role as pollinators, as supported by a recently published study.

## Introduction

Flies (Diptera) are the second most important group of flower-visiting insects world-wide [1]. Flies particularly in the sub-orders *Nematocera* and *Brachycera* are well known to visit flowers in search for nectar and pollen [1,2]. The common green bottle fly, *Lucilia sericata* (Meigen), and other calliphorid blow flies are well known to feed on carrion and feces but are also frequently observed on plant inflorescences [3–6]. Yet, the foraging cues they exploit to locate pollen- or nectar-producing plants, and the potential nutritional benefits of pollen and nectar on ovary development and maturation in flies have not yet been investigated.

To mature their oocytes, blow flies require large amounts of protein [7–9] which they obtain from various resources including carrion and animal feces [10,11]. Although of high quality, carrion protein is ephemeral and thus less dependable as a nutritional resource [12,13]. In comparison, animal feces has a low protein content (0.5–7%) [10,11,14–16] and, as such, represents a poor diet for the rapid maturation of fly oocytes prior to oviposition. Flower pollen, in contrast, has a protein content of 7% to 80% and contains low-molecular-weight proteins [17], which are essential for oocyte development [18,19]. Thus, pollen, as a resource, may be more reliable and possibly more suitable than animal feces.

Frequent protein meals are crucial for gravid blow fly females to develop and maintain their reproductively mature oocytes, which they otherwise resorb [16,20]. When protein-deprived females of the flesh fly *Sarcophaga bullata* Parker (Diptera: Sarcophagidae) are given a food choice between an amino acid-sugar mix and sugar, they feed on the former suggesting that these females detect the amino acids and exhibit a nutrition-based feeding preference [21,22]. In addition to the nutritional value of amino acids and proteins, flies require carbohydrates throughout their lives to fuel their daily activities and to increase their fecundity [23]. For example, carbohydrate-deprived females of the mosquito *Anopheles gambiae* Giles (Diptera: Culicidae) produce fewer and smaller eggs than well-fed mosquitoes [24]. Evidence that blow flies can digest both pollen protein as well as floral nectar supports the two concepts that blow flies visit plant inflorescences to obtain food and that they may become obligate foragers on floral pollen and nectar when animal feces and carrion are scarce. Expectedly then, calyptrate flies (like blow flies) are main pollinators in agricultural systems and are effective pollinators of some plant species [25,26].

Fly pollinators respond to both olfactory and visual cues of plant inflorescences [6, 27]. The attractiveness of inflorescences, though, seems to depend on the flies’ reproductive status and physiology. Inflorescences of sapromyophilous plants such as the dead horse arum lily, *Helicodiceros muscivorus* (Schott ex. K. Koch), often appear dark red and emit decomposition odorants including dimethyl disulfide and dimethyl trisulfide. These odorants are reminiscent of carrion odor [28,29], and thus are particularly attractive to gravid female blow flies seeking oviposition sites [30]. Unlike sapromyophilous flowers, myophilous flowers reward visitors with nectar, and possibly pollen, and produce a broad range of colors and typically sweet smelling fragrances [31]. The fragrances include monoterpenes, fatty acid-derived acids, alcohols, and nitrogen-containing compounds [27,32] but lack the distinct oligosulfide-dominated stench of sapromyophilous flowers [33].

Pollen- or nectar-foraging blow flies may exploit both the semiochemical and visual inflorescence cues that the myophilous flowers have to offer. While both *L*. *sericata* and the Australian sheep blow fly *L*. *cuprina* (Wiedemann) (Diptera: Calliphoridae) seem to have an innate affinity for yellow colors [34–36], it remains unknown whether they respond to yellow when they forage for nectar or pollen. It also remains unknown whether visual and semiochemical floral cues have interactive or synergistic effects on foraging decisions by blow flies. Such interactions are conceivable given that gravid *L*. *sericata* females respond well to a bimodal cue complex of dimethyl trisulfide and dark color, two cues which in combination apparently signify suitable oviposition sites such as fresh carrion [30,37–40].

*Lucilia sericata* was selected for this study because it is (*i*) commonly observed on flowers, (*ii*) frequently tested in the context of alternative pollination [5,6,41,42], (*iii*) geographically wide-spread, (*iv*) representative of other flies within the family calliphoridae [43,44], and (*v*) easily reared in the laboratory. As a model flower, the Oxeye daisy, *Leucanthemum vulgare* Lam., was selected because it offers a large pollen disc as a protein source for pollinators, and it is commonly visited by blow flies.

This study was undertaken to understand the effect of floral color and odor on foraging decisions by *L*. *sericata* females, and the effect of pollen protein on the ability of females to mature their oocytes. Experiments were conducted to determine whether pollen could possibly substitute animal feces or carrion as a protein source, which if so shown, would enhance the role of pollen as a food source for flies, and the role of flies as pollinators for composite flowers. The specific research objectives were to investigate (1) the effect of floral color cues on attraction of flies; (2) the effect of floral odor on attraction of flies; (3) potential interactions between visual and olfactory cues on attraction of flies; and (4) the effect of pollen on the ability of females to mature their oocytes.

## Materials and Methods

### Source of experimental flies and flowers

Flies were reared in the insectary of Simon Fraser University (SFU), starting a new colony with approximately 50 gravid wild-type flies every 12 months, and increasing the colony to about 5000 flies at specific times depending on experimental requirements. Flies were kept under a L16:D8 photoperiod, at 30–40% relative humidity, and a temperature of 23–25°C, and providing water only, with the exception of Objective 4 in which flies were provided a variety of food resources. Oxeye daisies were collected on the SFU campus where they are abundant from mid-April to June, facilitating their deployment in various experiments.

### General Design of Behavioral Experiments

**Fig 1:**
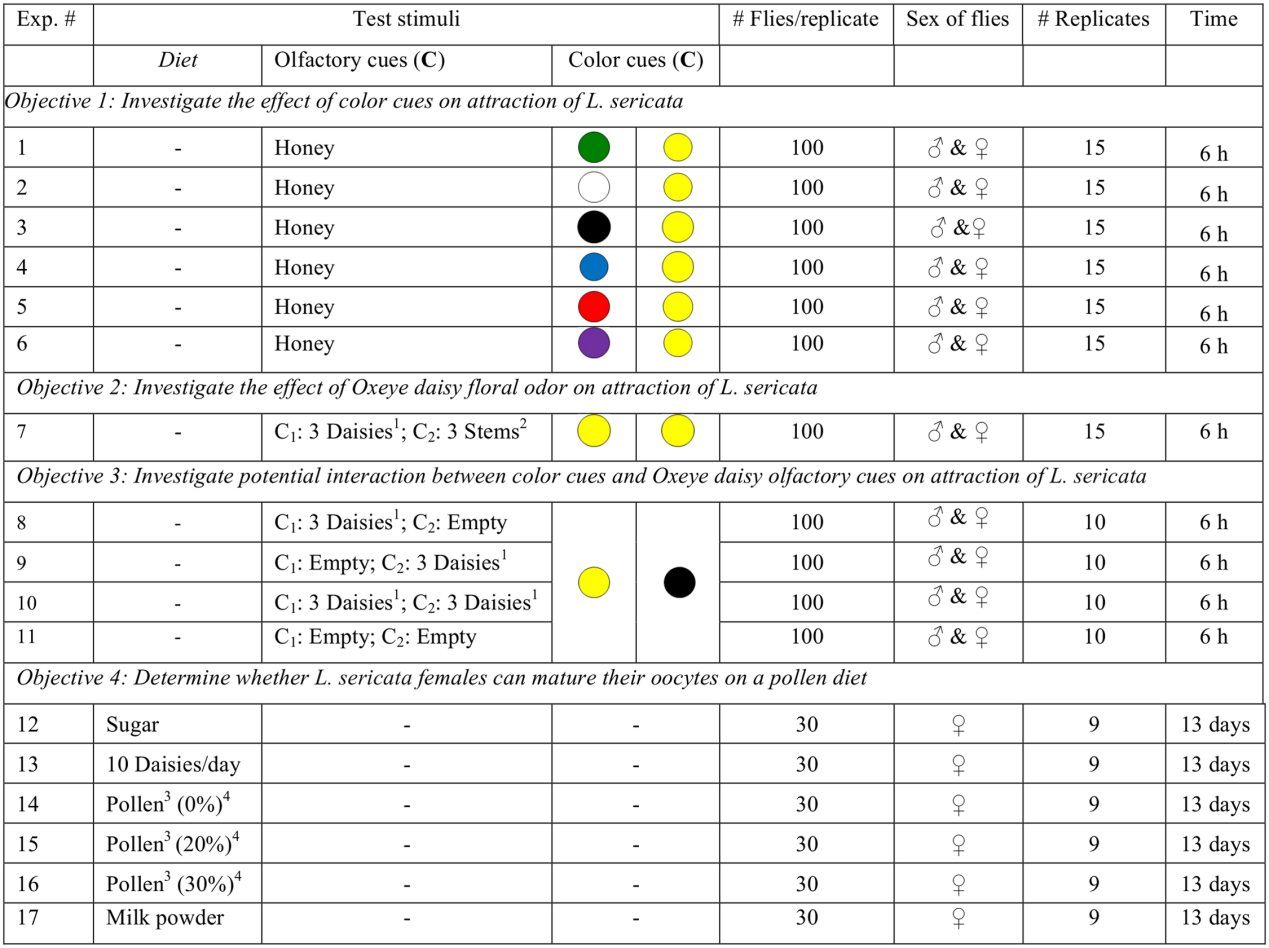
Details on the diet of experimental flies, olfactory and visual cues tested, numbers of flies tested per replicate, the duration of replicates, and numbers of replicates per experiment. ^1^Oxeye daisy inflorescence on 1-cm long stem; ^2^1-cm long stem without inflorescence; ^3^honey bee-collected pollen; ^4^% moisture content.

**Fig 2:**
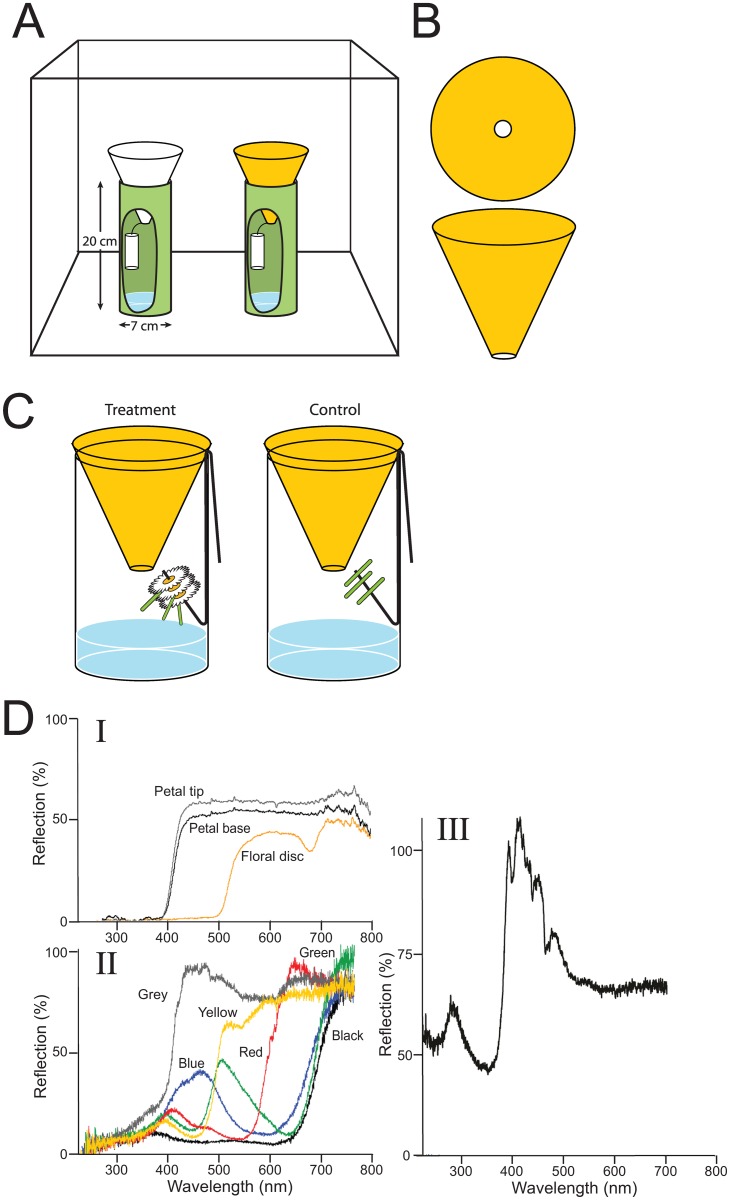
Graphical illustrations of experimental designs. (A, B) Design of two-choice laboratory experiments (see Fig 1) with inverted bottle traps (see methods for detail), consisting of a green trap base and a funnel-like trap top covered with paper of a particular test color, and baited with honey (A), or with three freshly-cut Oxeye daisy inflorescences on 1-cm long stems or three corresponding stems (C) as the olfactory cues; (D) representative (*n* = 5 each) spectral reflectance profiles from (**I**) Oxeye daisy inflorescences [floral disc (yellow), petal tip (grey), petal base (black)], (**II**) yellow, white, red, blue, green, or black construction papers tested in color choice experiments, and (**III**) UV-reflective paper; the color of each reflectance curve in **I-III** corresponds to the color of the material measured; in **I** and **III**, black curves represent UV reflections.

The response of flies to visual and olfactory stimuli was tested in 2-choice experiments 1–11 (Fig 1) using BioQuip^®^ (Compton, CA) wire mesh cages (61 × 61 × 61 cm) with a plated grey base (BioQuip^®^, Compton, CA, USA). Each cage was illuminated from above with fluorescent lights (Phillips F32TA, Amsterdam, The Netherlands; light intensity in cage: 236 Lux). For each experimental replicate, 100 recently eclosed (1- to 3-day-old) cold-sedated flies of mixed sex (approximate sex ratio 50:50) were introduced into a cage, allowing them to acclimate for 2 h prior to the start of the experiment. The stimuli was tested as part of an inverted “bottle trap” consisting of a 500-mL plastic soda bottle with the inverted top providing a cone-shaped funnel (11.5 cm long × 0.6 cm bottom diameter × 7.9 cm top diameter; Fig 2A) that rested on the bottom half (20 cm long × 7 cm diameter; Fig 2A). The funnel was covered with construction paper (11.5 cm long × 0.6 cm diameter) of a specified color as the visual test cue (Fig 2D) and wrapped the bottom half with green construction paper (Fig 2A), randomly assigning test stimuli to opposite corners of the bioassay cage. A mote of Sparkleen^™^ (Fisher Scientific Co. Pittsburgh, PA, USA) and water (0.5:5) in the trap bottom drowned the flies that entered the trap. Trap captures were scored after a 6-h experimental duration.

### Objective 1 (experiments 1–6): Investigate the effect of color cues on attraction of *L*. *sericata*

To test whether flower-foraging flies respond best to a bimodal complex of odor and visual (color) cues, but to nevertheless isolate the effect of color cues, odor was standardized by using 2 g of honey (concentrated nectar) (Wedderspoon Organic Inc., Dancan, B.C., Canada) as a generic floral odor source and was placed in a 20-mL vial suspended by wire inside the inverted bottle traps (Fig 2A). Honey odorants emanated from the vial through a mesh-covered hole (1.5-cm diameter) in the lid that prevented flies from accessing the honey, which is attractive to young *L*. *sericata* (Brodie et al., unpubl. data). The visual test color cues was standardized by shaping the construction paper such that it fit perfectly over the surface of the funnel-shaped trap top (Fig 2B).

In each of parallel-run experiments 1–5 (Fig 1), we tested the effect of a uniformly yellow cue *versus* that of a uniformly green, white, black, blue, or red cue [all color cues: SunWorks^®^ construction paper (Pacon Corporation, Appleton, WI, USA)]. Parallel-run experiment 6 (Fig 1) had the same design, but a different type of paper (Husky^®^ copy30; Domtar, Montreal, QC, Canada) was deployed to generate ultra-violet (UV) light reflections. UV reflections are inflorescence cues known to attract bee and syrphid fly pollinators and to play a role as floral guides [45,46].

Spectrometric profiles (*n* = 5 each) was measured for all paper-derived color cues and the spectrometric profiles of the daisies’ petal tips, petal base, and floral disc using an Ocean Optics Inc. spectrometer (Dunedin, FL, USA).

### Objective 2 (experiment 7): Investigate the effect of Oxeye daisy floral odor on attraction of *L*. *sericata*

A binary choice assay was used to investigate whether only generic honey odor, but also the specific inflorescence odor of Oxeye daisies, attracts *L*. *sericata* in the presence of a key visual cue (yellow) (Fig 1). To this end, the funnel tops of each of the two traps were fitted with yellow construction paper and randomly assigned three freshly cut Oxeye daisy inflorescences with a 1-cm long stem each to one trap and three equivalent 1-cm long stem sections with the inflorescence severed to the other trap (Fig 2C). The plant material was suspended by piercing a wire through the plant tissue, and attached to the funnel top of the trap (Fig 2C).

### Objective 3 (experiments 8–11): Investigate potential interactions between color cues and Oxeye daisy inflorescence olfactory cues on attraction of *L*. *sericata*

In binary choice experiments 8–11 (Fig 1), potential interactions were tested between visual and olfactory cues on attraction of *L*. *sericata*. Floral odor cues were generated by suspending three freshly cut Oxeye daisy inflorescences inside the trap (see above; Fig 2C), and visual cues were generated by covering the trap funnel with either yellow- or black-colored construction paper (see above), while invariably covering each trap bottom with green-colored construction paper. Yellow was selected as a visual test cue because it is particularly attractive to young *L*. *sericata* females (see Results) that may be foraging for floral nectar and pollen (see below). Black was selected because it is less attractive to young females (see Results) but is part of a bi-modal cue complex (with dimethyl trisulfide) that is particularly attractive to gravid *L*. *sericata* females seeking oviposition sites [30]. Using a factorial design, the combinations specifically tested included: (1) yellow with Oxeye daisy *versus* black with Oxeye daisy (Exp. 8); (2) yellow alone *versus* black alone (Exp. 9); (3) yellow alone *versus* black with Oxeye daisy (Exp. 10); and (4) yellow with Oxeye daisy *versus* black alone (Exp. 11). To avoid carry-over effects of Oxeye daisy odor, experiment 9 (which tested the effect of just visual cues) was conducted in a second bioassay room with identical temperature and relative humidity conditions as the first bioassay room. Experiments 9–11, with 2- and 3-day-old flies, were run with an equal number of replicates for each experiment on both days.

### Objective 4 (experiments 12–17): Determine whether *L*. *sericata* females can mature their oocytes on a pollen diet

In Experiments 12–17 (Fig 1), the effect of a specific food source on the ability of *L*. *sericata* females to mature their oocytes was tested as follows: (1) granulated sugar (negative control); (2) Oxeye daisy pollen on 10 freshly cut inflorescences; (3–5) honey bee-collected pollen (The Honey Bee Centre, Surrey, BC, Canada) with a moisture content of 0% (3), 20% (4) and 35% (5); and (6) milk powder (positive control). For treatment 2, we collected Oxeye daisies on the SFU campus and replaced them every day before the onset of the photophase. To produce the three levels of pollen moisture content, honey bee-collected pollen was placed in a desiccator and weighed daily until there was no further weight reduction. We then finely ground the dry pollen for 5 min in a coffee grinder (Black and Decker; Towson, Maryland, USA) after which distilled water was added to produce pollen with 20-% or 35-% moisture content. As moisture content of pollen changes over time due to water evaporation, pollen in treatments 3–5 was replaced every second day.

For each experiment, 30 recently eclosed female flies were placed in a wooden-framed cage with nylon mesh (25 cm high × 15 cm × 15 cm), which were allowed to feed on the food source *ad libitum* for 13 consecutive days. Flies were then freeze-killed and stored in the freezer for later dissection. To assess the food effect on the maturation of oocytes, we removed the oocytes, placed them in a drop of Ringer’s saline on a microscope slide, and scored 10 phases of follicle development according to the scheme of Adams & Reinecke [47] for the primary screwworm, *Cochliomyia hominivorax* (Coquerel). As previously described [11], phases of follicle development were grouped into three main stages: (I) phases 0–3: oocytes with dividing cells; (II) phases 4–9: oocytes with yolk sac, and (III) phase 10: mature and chorionated eggs. In addition, flies were randomly selected to examine the ventriculus content for evidence of pollen.

### Statistical analyses

We analyzed data from experiments 1–7 (objectives 1, 2: test for attraction of flies to color (UV) cues and Oxeye daisy odor cues, respectively; see Fig 1) with a non-parametric Wilcoxon signed rank test. For experiments 8–11 (objective 3: test for interactions between color cues and Oxeye daisy odor cues on attraction of flies), we used generalized linear models with a Poisson distribution, corrected for overdispersion, and used the number of fly captures as the predicted variable and *odor*, *visual cue* and the interaction term *odor × visual cue* as the predictor variables (see Fig 1). For each of experiments 1–11, we also used a chi-square goodness of fit test to analyze potential effects of floral color, floral odor, or color and odor interactions, on the sex ratio of responding flies. For experiments 12–18 (objective 4: test for effect of diet on fly ovary development), we used a logistic regression to test whether ovary development was dependent on diet. First, the proportion of oocytes at each of the three developmental stages (I, II, III) was used as the predicted variable, and *diet* (factor with 6 levels), *developmental stage* (factor with 3 levels), and the interaction term *diet × developmental stage* as predictor variables. Second, separate logistic regression analyses were used for each developmental stage, using the proportion of oocytes at that particular stage as the predicted variable, and diet as the predictor variable. Lastly, we performed pairwise comparisons between diets to identify the effect of diet on ovary development using the Tukey HSD adjusted least square means test. All statistical analyses were produced with JMP 10^®^ (SAS Institute Inc.) for Windows^®^ (Windows Corporation, Redmond, WA, USA).

## Results

### Objective 1 (experiments 1–6): Investigate the effect of color cues on attraction of *L*. *sericata*

**Fig 3:**
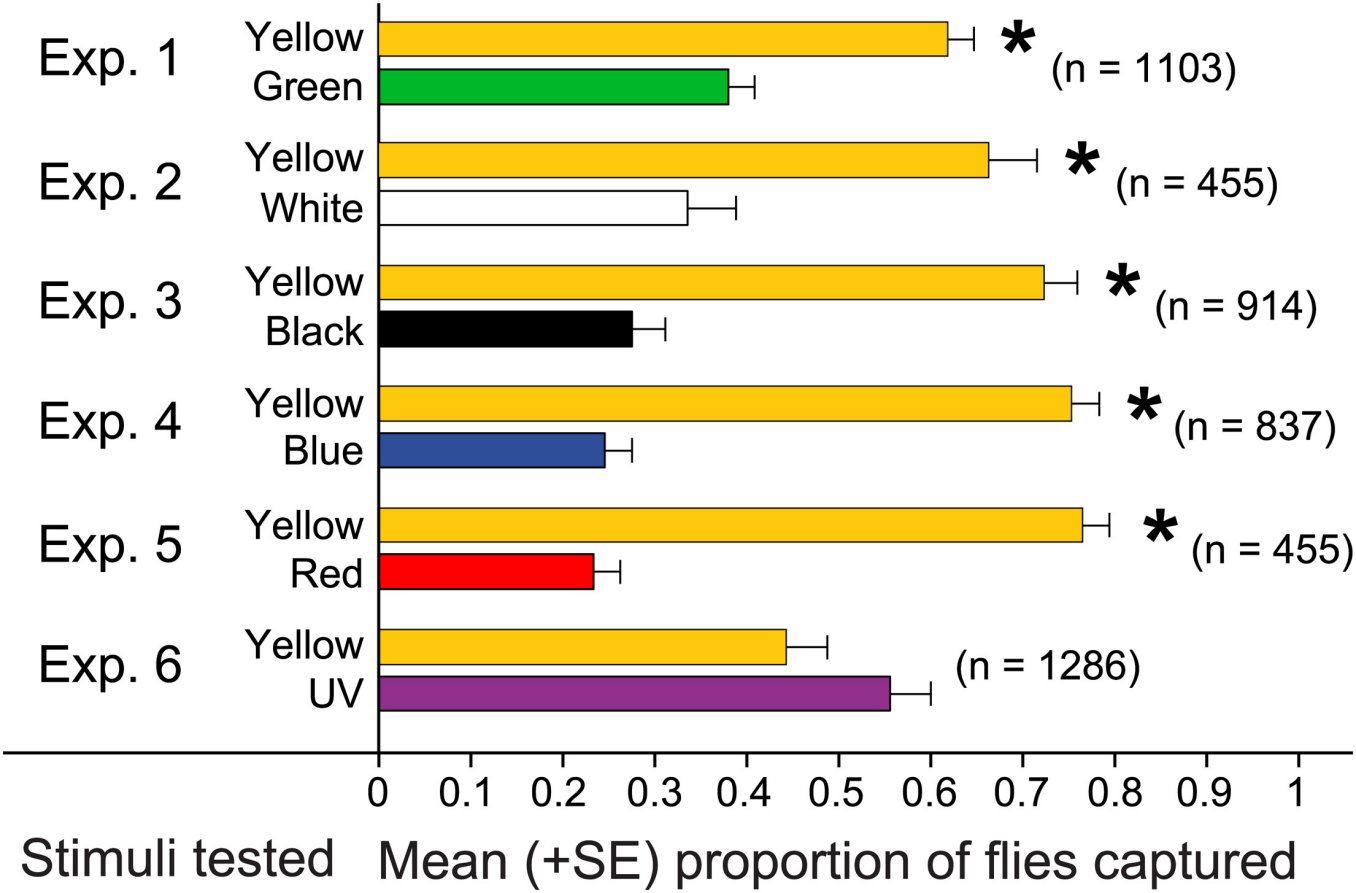
Effect of color cues on attraction of *Lucilia sericata*. Mean proportions of 1-, 2-, and 3-day-old males and females captured in experiments 1–6 (*n* = 15 each; Fig 1) in inverted bottle traps (Fig 2A) that were baited with a generic floral scent (honey) and with a specific color cue (Fig 2D) covering the inner surface of the trap funnel. In each experiment, the number in parenthesis indicates the total number of flies captured, and an asterisk (*) on a bar indicates a significant preference for the test stimulus (Wilcoxon signed rank test, *p* < 0.05).

Yellow had a significant effect on captures of flies (Fig 3; exps. 1–5). Traps with a yellow top captured significantly more flies than traps with a red top (Z = 4.65, df = 1, *p* = < 0.0001), blue top (Z = 4.65, df = 1, *p* < 0.0001), green top (Z = 4.06, df = 1, *p* < 0.0001), white top (Z = 3.45, df = 1, *p* = 0.0006) or black top (Z = 4.48, df = 1, *p* < 0.0001). In contrast, traps with a yellow top were not significantly more effective in capturing flies than traps with a UV light-reflecting top (Exp. 6: Z = 0.00, df = 1, *p* = 1).

For each of experiments 1–6, there was no significant difference in the sex ratio of flies captured (*p* > 0.05).

### Objective 2 (experiment 7): Investigate the effect of Oxeye daisy floral odor on attraction of *L*. *sericata*

**Fig 4:**
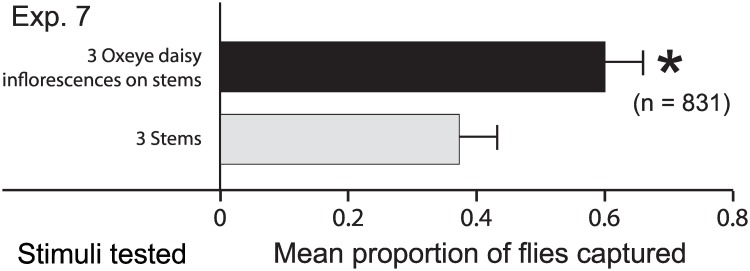
Effect of floral odor on attraction of *Lucilia sericata*. Mean proportion of 36-h-old, females and males captured in experiment 7 (*n* = 15; Fig 1) in two inverted bottle traps (Fig 2A and 2B) with yellow trap funnels that were baited with either three freshly-cut Oxeye daisy inflorescences on 1-cm long stems or three corresponding stems (Fig 2C). The number in parenthesis indicates the total number of flies captured, and the asterisk (*) indicates a significant preference for the test stimulus (Z = -2.95, df = 1, *p* = 0.0028).

Traps baited with Oxeye daisy inflorescences on 1-cm stems captured significantly more flies than traps baited with 1-cm stems alone (Fig 4; Z = -2.95, df = 1, *p* = 0.0028). There was no significant difference in the sex ratio of flies captured (*p* > 0.05)

### Objective 3 (experiments 8–11): Investigate potential interactions between color cues and Oxeye daisy inflorescence olfactory cues on attraction of *L*. *sericata*

**Fig 5:**
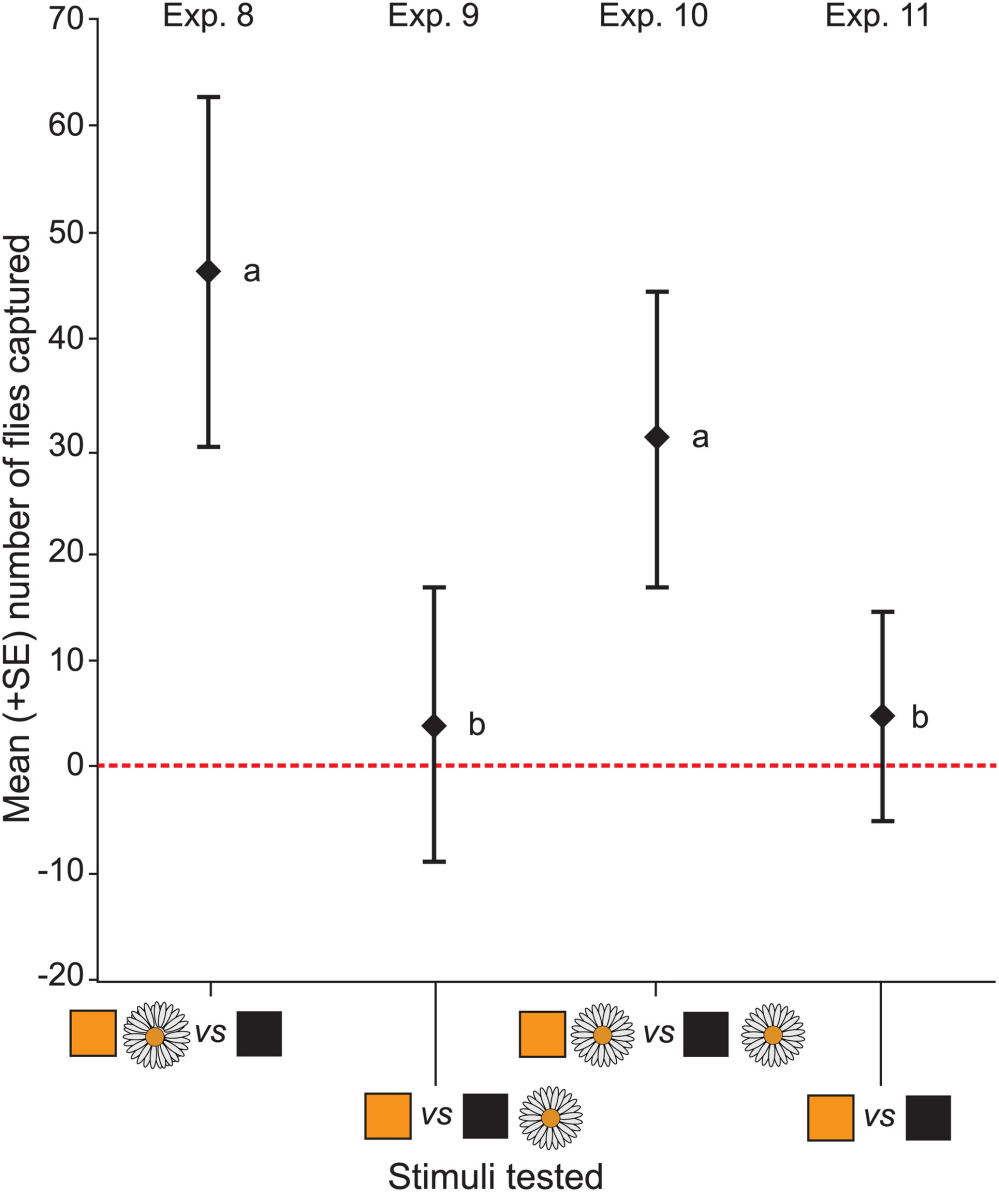
Interactions between visual and olfactory cues on attraction of *Lucilia sericata*. Mean number of females and males captured in experiments 8–11 (*n* = 10 each; Fig 1) in paired bottle traps (Fig 2A and 2B) baited with the following cue combinations: Exp. 8: Yellow with Oxeye daisy inflorescence (Fig 2C) *versus* Black with Oxeye daisy inflorescence; Exp. 9: Yellow alone *versus* Black alone; Exp. 10): Yellow alone *versus* Black with Oxeye daisy inflorescence; and (Exp. 13): Yellow with Oxeye daisy inflorescence *versus* Black alone. Replicates of all experiments were run in parallel but those for experiment 9 (which tested the effect of color only) were run in a separate room. Flies significantly preferred traps with yellow funnel tops (*χ* ^*2*^_*1*_ (1, *N* = 40) *= 22*.*83*, *p* < 0.001) and traps baited with Oxeye daisy inflorescence odor (*χ* ^*2*^_*1*_ (1, *N* = 40) = 22.8, *p* = 0.003) but there was no interaction between color and odor (*χ* ^*2*^_*1*_ (1, *N* = 40) = 0.018, *p =* 0.894).

The color of the trap top funnel (*χ* ^*2*^_*1*_ (1, *N* = 40) *= 22*.*83*, *p* < 0.001) and daisy odor (*χ* ^*2*^_*1*_ (1, *N* = 40) = 22.8, *p* = 0.003) both had a significant effect on the number of flies captured (Fig 5). Captures of flies increased in the presence of a yellow trap top or daisy odor, but there was no interaction between the color yellow and daisy odor (Fig 5: *χ* ^*2*^_*1*_ (1, *N* = 40) = 0.018, *p =* 0.894). For each of experiments 8–11, there was no significant difference in the sex ratio of flies captured (*p* > 0.05).

### Objective 4 (experiments 12–17): Determine whether *L*. *sericata* females can mature their oocytes on a pollen diet

**Fig 6:**
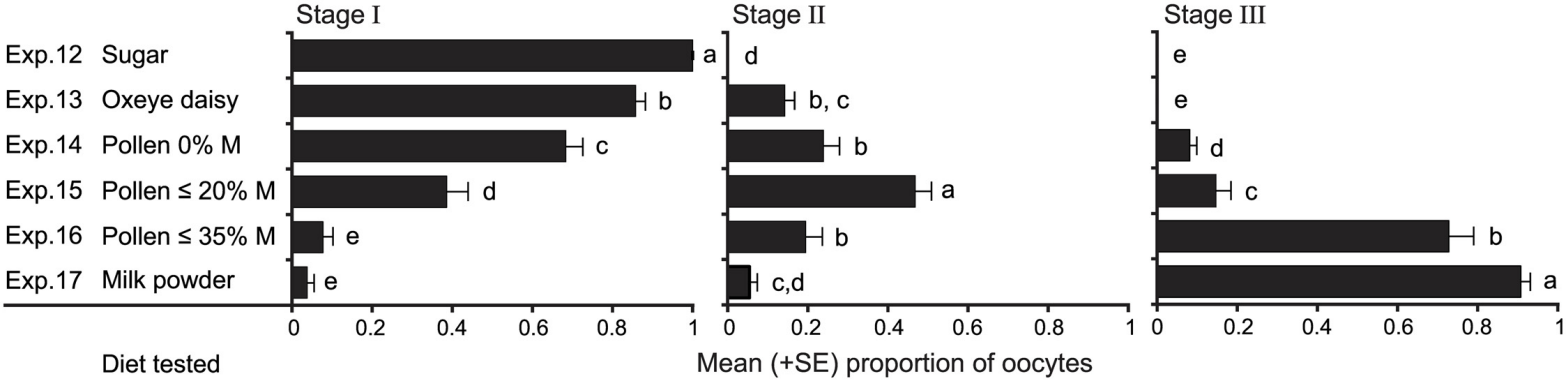
Effect of diet on the ability of *Lucilia sericata* females to mature their oocytes. To assess the effect of diet [sugar (negative control; Exp.12), Oxeye daisy pollen from fresh inflorescences (Exp. 13), honey bee-collected pollen with 0%, ≤20% or ≤35% moisture content (Exps. 14–16), and milk powder (positive control, Exp. 17) (*n* = 9 each; Fig 1) on the ability of *L*. *sericata* females to mature their oocytes, we scored 10 phases of ovary development according to Adams & Reinecke (47) and grouped phases into three main stages: (I) phases 0–3: oocytes with dividing cells; (II) phases 4–9: oocytes with yolk sac, and (III) phase 10: mature and chorionated eggs. Diet had a significant effect on ovarian maturation (*F*_2, 147_ = 153.62, *p* < 0.0001). Within each of stage I, II and III, bars with different letters indicate significant differences in the mean proportions of fly oocytes at that stage based on diet (Tukey’s HSD: *p* <0.05).

In randomly selected flies, we observed broken pollen grains in their gut. Females matured their oocytes to various degrees based on the type and moisture content of pollen. There was a significant interaction between food type and ovarian stage (*F*_17, 147_ = 117.98, *p* <0.0001, Fig 6). Stage-I oocytes were present in 33%, 28% and 22% of flies that had fed on sugar, Oxeye daisy pollen and 0% moisture content bee-collected pollen, respectively (Fig 6). Stage-II oocytes were present in 22% of flies that had fed on bee-collected pollen with ≤20% moisture content. Lastly, stage-III oocytes were present in 48% and 39% of flies, respectively, that had fed on milk powder or bee-collected pollen with ≤35% moisture content (Fig 6).

## Discussion

This study contributes to the current understanding of how and why blow flies forage for flowers, with *L*. *sericata* as the model fly species, and the Oxeye daisy as the model plant species among the composite flowers that blow flies so commonly visit [3–6]. Our data indicate that (1) young flies respond more strongly to a uniformly yellow cue than to any other uniform color cue (green, white, black, blue, red) except UV; (2) floral odor of Oxeye daisies enhances the attractiveness of yellow; and (3) pollen provides nutrients that contribute to ovary maturation of flies. Below we discuss the implications of our findings.

Of the many species of flies that are capable of color vision [48–50], the Calliphoridae (blow flies), Syrphidae (hover flies), Tephritidae (fruit flies) and Anthomiidae (house flies) innately prefer yellow [35, 51–54]. Our data corroborate the results of previous reports that blow flies prefer yellow, at least when flies are young and in flower-foraging mode. As expected, flower-foraging *L*. *sericata* females also respond well to UV, likely because the inflorescences of their commonly visited plants in the Apiaceae and Asteraceae often provide UV floral guides [45,55]. That uniformly blue or red colors were least attractive to flies (Fig 3; experiments 4, 5) may be attributed to evidence that these colors attract other pollinators and thus help partition floral resources among floral visitors [51,56,57] that compete for nectar and pollen. Indeed, bumblebees primarily visit inflorescences with dark blue or violet (long) petals, whereas flies primarily visit inflorescences with yellow or white (short) petals. Expectedly then, plant species such *Raphanus raphanistrum* L. and *Myosotis sylvatica* Ehrh. (Family: Brassicaceae) with multiple floral color morphs [58], or floral color changes over time [59], attract both fly and bee pollinators.

Floral odor alone or in conjunction with floral color attracts insects [60,61] and plays a significant role in plant-pollinator interactions. Changes in floral odor alter its attractiveness to pollinators [62]. For example, the omission of sulfur-containing odorants from floral odor mediated a shift from fly to wasp pollinator systems [63]. Yet, blow flies are observed on the inflorescences of diverse plant species, many of which are not producing sulfur-containing or necromyophylous odorants [1,32,64]. In this study, the sweat-like odor of Oxeye daisies clearly affected the behavior of flies (Fig 4) but their responses were dependent upon the presence of a specific color cue (Figs 3 and 5). Trap pairs with yellow or black funnel tops, each coupled with daisy odor, captured significantly more flies than the same trap pairs lacking daisy odor (Fig 5). Similarly, when only one of the paired traps was coupled with daisy odor, the pair presenting a combination of yellow and daisy odor induced more fly captures than the pair presenting a combination of black and daisy odor (Fig 5). The ineffectiveness of the latter combination could be explained by two factors. Firstly, the mixed message that we deliberately presented. Naturally, daisy odor accompanies yellow rather than black floral cues. In contrast, carrion smell typically accompanies the dark pelt of the recently deceased animal. Simply put, there is no such thing as dark carrion smelling like daisy. Secondly, the physiological stage of flies alters their foraging response and preference [65, 66]. Recently eclosed flies need protein and carbohydrates to mature their oocytes [16,67] and respond well to a bi-modal cue complex of floral color (e.g., yellow) and floral odor (e.g., daisy odor) that is indicative of pollen protein and nectar (this study). Gravid flies, in turn, that seek oviposition sites respond better to a cue complex of carrion odor and dark color than to a complex of carrion odor and white or yellow color [30]. The above examples indicate not only that fly foraging decisions are affected by bi-modal cues more strongly than a mono-modal cue, they also indicate that combination preference change in accordance with the physiological stage of the flies. That flies respond to distinctively different and to bi- or multi-modal cue complexes when they forage for floral resources and oviposition sites, respectively, appears to be reflected in the type of cues that two sympatric plant congeners present to pollinators. The pale yellow and sweet-smelling inflorescences of *Metrodorea stipularis* Mart. likely appeal to protein- and nectar-foraging flies, whereas the violet-brown disagreeable-smelling inflorescences of *Metrodorea nigra* St. Hill. likely attract gravid flies [68].

*Lucilia sericata* females are indeed capable of digesting flower pollen. Broken pollen grains were observed in the gut of flies and recorded at least partial maturation of oocytes in flies on a pollen diet (Fig 6). This is noteworthy because few animals, including adult insects, can efficiently digest pollen [69]. The pollen walls are apparently difficult to penetrate or dismantle. Insects gain access to the nutrient-rich cytoplasm in the center of pollen grains by inducing germination or pseudo-germination [70–72], bursting the pollen walls through osmotic shock [73], and by penetrating the pollen walls with digestive enzymes [69]. Flies may pre-treat pollen with saliva for easier uptake or pre-digestion, and may pressure it with their proventriculus in the digestive tract that can break cell walls and thus facilitate access to the nutrient-rich cytoplasm. Given the importance of microorganisms to blow flies[74–76], it is conceivable that bacteria in the saliva and digestive tract of blow flies [77,78] assist in the breakdown of pollen.

The ability of *L*. *sericata* females to digest pollen increased with the moisture content of pollen (Fig 6), which averages 20% among plant species [79–80]. The sponging mouthparts of flies may be more capable of handling moist pollen than dry pollen, and the former may be more easily processed through the digestive tract. Unexpectedly, our experimental flies could not mature their oocytes to stage III on Oxeye daisy pollen alone (Fig 6). Advanced ovary development may require specific pollen from multiple plants, which would explain why blow flies visit the inflorescences of many plant species [4–6], and why the bee-collected pollen with its diverse nutrient composition provided a diet suitable for ovary development to stage III (Fig 6).

In summary, this data supports the conclusion that flower-foraging *L*. *sericata* respond to a bi- or even multi-modal cue complex comprising at minimum both floral odor and specific floral colors (including UV). Female flies are attracted to plant inflorescences because pollen proteins, and nectar [81], provide nutrients that help flies mature their oocytes. With evidence that floral protein can serve as an alternate or supplement to animal feces or carrion protein, pollen may play a major role in the foraging ecology of *L*. *sericata* and possibly other filth flies that, in turn, then may play a significant role as pollinators [26]. This concept is supported by evidence that blow flies are highly effective at single pollen deposition for a variety of flowers [25], which is a good proxy for successful pollination.

## Supporting Information

S1 Dataset Experimental data (Exps. 1–17) on blowfly attraction to flower cues and oocyte development on pollen diet. (XLSX)
